# Sensory hair cell death and regeneration in fishes

**DOI:** 10.3389/fncel.2015.00131

**Published:** 2015-04-21

**Authors:** Jerry D. Monroe, Gopinath Rajadinakaran, Michael E. Smith

**Affiliations:** ^1^Department of Biology, Western Kentucky UniversityBowling Green, KY, USA; ^2^Department of Genetics and Genome Sciences, University of Connecticut Health CenterFarmington, CT, USA

**Keywords:** hair cell, regeneration, ototoxicity, zebrafish, cell death, teleost, acoustic trauma, lateral line

## Abstract

Sensory hair cells are specialized mechanotransductive receptors required for hearing and vestibular function. Loss of hair cells in humans and other mammals is permanent and causes reduced hearing and balance. In the early 1980’s, it was shown that hair cells continue to be added to the inner ear sensory epithelia in cartilaginous and bony fishes. Soon thereafter, hair cell regeneration was documented in the chick cochlea following acoustic trauma. Since then, research using chick and other avian models has led to great insights into hair cell death and regeneration. However, with the rise of the zebrafish as a model organism for studying disease and developmental processes, there has been an increased interest in studying sensory hair cell death and regeneration in its lateral line and inner ears. Advances derived from studies in zebrafish and other fish species include understanding the effect of ototoxins on hair cells and finding otoprotectants to mitigate ototoxin damage, the role of cellular proliferation vs. direct transdifferentiation during hair cell regeneration, and elucidating cellular pathways involved in the regeneration process. This review will summarize research on hair cell death and regeneration using fish models, indicate the potential strengths and weaknesses of these models, and discuss several emerging areas of future studies.

## Introduction

Hair cells are specialized mechanosensory receptors which convert external vibratory stimuli into neural signals (Hudspeth et al., 2000; Hackney and Furness, 2013). These cells are present in vertebrate auditory and vestibular organs and the lateral line system of aquatic vertebrates (Hudspeth, 1989; Corwin and Warchol, 1991; Nicolson, 2005). Hair cells can be damaged or destroyed by acoustic or chemical exposure, but non-mammalian hair cells, unlike their mammalian counterparts, can regenerate after they are damaged or lost (Corwin and Oberholtzer, 1997; Brignull et al., 2009; Rubel et al., 2013). This regenerative ability has sparked interest in studying hair cell death and restoration in fish in order to find means of promoting sensory hair cell regeneration in humans.

Ototoxic chemicals such as heavy metals, aminoglycoside antibiotics and platinum containing drugs like cisplatin that damage mammalian hair cells also target fish lateral line and inner ear hair cells (Lombarte et al., 1993; Ton and Parng, 2005; Hernández et al., 2006; Olivari et al., 2008; Giari et al., 2012; Uribe et al., 2013a). As in other vertebrates, auditory hair cells in fishes can also be damaged by intense acoustic stimuli (Smith et al., 2006; Schuck and Smith, 2009; Casper et al., 2013). Hair cell loss in fishes causes hearing and vestibular deficits (Corwin and Oberholtzer, 1997; Matsui and Ryals, 2005; Rubel et al., 2013) which can be assessed via behavioral and electrophysiological methods (Smith et al., 2006; Suli et al., 2012; Ladich and Schulz-Mirbach, 2013).

As mammalian and fish hair cells are damaged by similar chemical and acoustic insults, many researchers are interested in studying the mechanisms of hair cell death and otoprotection in fish with the objective of developing therapeutics that may treat or prevent human hair cell loss. A majority of this research has been done in zebrafish (*Danio rerio*), which has become an important vertebrate model for examining embryogenesis, organ development, disease, and genetic defects (Kimmel, 1989; Driever et al., 1996; Haddon and Lewis, 1996; Zon, 1999) and is also a valuable model in hearing research. For example, the zebrafish mutants mariner and sputnik, which do not respond to vibrational stimuli, have hair cell bundle defects where the stereociliary bundles are detached from the kinocilia and are splayed (Nicolson et al., 1998). Genetic screens using zebrafish inner ear mutants have identified alteration of ear morphology, including expansion of the ear lumen, and aberrant formation and shape of the otolith (Malicki et al., 1996; Schibler and Malicki, 2007). Further, zebrafish mutants can exhibit morphological and functional defects similar to those of mouse mutants (Ernest et al., 2000). Considerable genetic and organ system homology exists between zebrafish and humans (Barbazuk et al., 2000; Goldsmith and Jobin, 2012). Many of the genes expressed in the embryonic zebrafish inner ear during the onset of hair cell differentiation and innervation are also expressed in other vertebrates (Coimbra et al., 2002). These include members of the Notch signaling pathway which regulate sensory cell commitment in mammalian models as well as numerous other orthologs with human genes. Thus, zebrafish models can provide insight into human hearing diseases and their mechanisms and have some advantages over mammalian models (Table 1).

**Table 1:** **Comparison of some advantages and disadvantages of zebrafish and rodent models of sensory hair cell death and regeneration**.

	Zebrafish		Rodent
Low husbandry cost/space per animal	+	Higher husbandry cost/space per animal	−
Large # of synchronous developing embryos per mating	+	Production of 3–14 pups/litter	−
Embryos and larvae translucent for ex utero visualization	+	Embryos opaque and *in utero*	−
Easier access of sensory hair cells	+	Hair cells embedded in bony capsule	−
Hearing range lower frequency than humans	−	Hearing range comparable to humans	+
Large-scale mutagenesis	+	Several years to develop knockout mice	−
High-throughput genetic/drug screening	+	Lower-throughput genetic/drug screening	−
*In vivo* model using neuromast hair cells	+	Inner ear must be dissected out	−
Transgenic models available	+	Transgenic models available	+
70% homology with human genome*	−	90% homology with human genome*	+

+/− Relative advantage/disadvantage. *Percentages are approximations.

Both hair cell damage and regeneration research in fishes have primarily focused on the lateral line because of its accessibility and visibility, which greatly facilitates the study of cellular and molecular mechanisms of development and differentiation (Brignull et al., 2009; Groves, 2010; Esterberg et al., 2013; Lush and Piotrowski, 2014a; Thomas et al., 2015a), although, recent efforts have begun to characterize these processes in the teleost inner ear as well (Schuck and Smith, 2009; Millimaki et al., 2010; Tanimoto et al., 2011). Researchers are also beginning to elucidate the genetic and molecular signaling mechanisms that regulate fish auditory sensory hair cell loss and restoration (Brignull et al., 2009; Ma and Raible, 2009; Schuck et al., 2011; Namdaran et al., 2012). This article reviews the current knowledge regarding hair cell death and regeneration in fishes, the advantages and disadvantages of using fish in these studies, and concludes by suggesting several future avenues for study.

## Anatomy and Physiology of Teleost Hair Cells

### The Teleost Lateral Line

**Figure 1:**
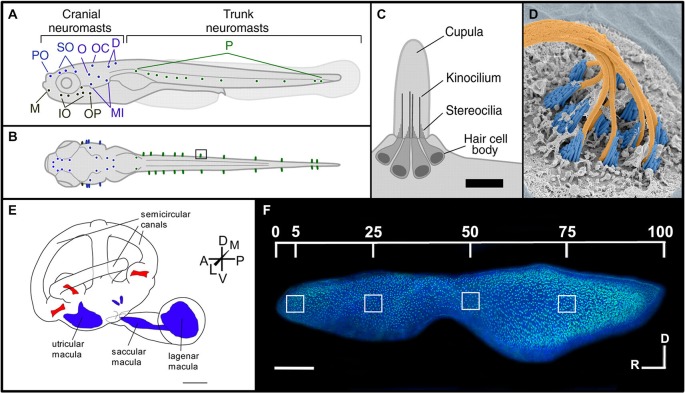
**Morphology of the lateral line and inner ear systems of zebrafish. (A)** Lateral and **(B)** dorsal views of zebrafish larvae illustrate the distribution of neuromasts along the body. The supraorbital region (blue) includes the preoptic (PO) and supraorbital (SO) neuromasts. The infraorbital region (black) includes the mandibular (M), infraorbital (IO) and opercular (OP) neuromasts. The caudal–cranial region (purple) includes the otic (O), occipital (OC), dorsal (D) and middle (MI) neuromasts. Finally, the posterior (P) neuromasts are located in the trunk region (green). **(C)** The morphology of an individual neuromast illustrates its major anatomical features. Scale bar, 10 μm. Reproduced with permission from Van Trump and McHenry (2008) **(D)** False-colored SEM image of a zebrafish neuromast. The cupula has been removed to see the kinocilia (orange) and the stereocilia (blue). Reprinted from cover image of Developmental Cell, Vol. 23, (2), K.S. Kindt, G. Finch, T. Nicolson, Kinocilia mediate mechanosensitivity in developing zebrafish hair cells, 2012, with permission from Elsevier. **(E)** Morphology of the zebrafish inner ear, showing the relative positions of the sensory maculae (blue) and cristae (red). Scale bar, 500 μm. Adapted with permission from Hammond et al. (2010). **(F)** Phalloidin- and DAPI-labeled zebrafish saccule, showing locations used for quantifying hair cell bundle densities along the rostral-caudal axis. Scale bar, 100 μm. Reprinted from Hearing Research, Vol. 253, J.B. Schuck, M.E. Smith, Cell proliferation follows acoustically-induced hair cell bundle loss in the zebrafish saccule, 2009, with permission from Elsevier.

The fish lateral line contains hair cells in sensory patches known as neuromasts (Figures 1A–D). These patches are arrayed either on the surface (superficial neuromasts) or in a fluid-filled channel with pores connecting to the outside (canal neuromasts) (Coombs et al., 2001; McHenry and van Netten, 2007). Neuromasts ultimately develop 15–20 hair cells surrounded by two accessory cell types, supporting and mantle cells (Villegas et al., 2012). They are classified as being either part of the anterior lateral line (ALL), which are located on the head, or the posterior lateral line (PLL), which are found along the trunk and tail (Ghysen and Dambly-Chaudière, 2004). Lateral line hair cells detect hydrodynamic pressure variations providing crucial sensory information required for fundamental behaviors including rheotaxis, schooling, prey detection, and predator avoidance (Coombs et al., 2001; Liao, 2006; Suli et al., 2012). Neuromast cellular layers and nervous connections are similar to those of the inner ear sensory macula (Nicolson, 2005; Haehnel et al., 2012). However, the ciliary bundles of lateral line hair cells project into a gelatinous compartment, the cupula, where vibrational forces are transduced by the gel’s motion against the ciliary bundles (Nicolson, 2005; McHenry and van Netten, 2007). Morphological observations show that newly differentiating or functionally immature hair cells mainly originate from the edge of the neuromast and migrate toward the center (Williams and Holder, 2000; López-Schier and Hudspeth, 2006; Kindt et al., 2012).

Most of what is known about hair cell death, regeneration and ototoxicity in fishes come from studies of zebrafish lateral line neuromasts (Williams and Holder, 2000; Harris et al., 2003; Hernández et al., 2006; López-Schier and Hudspeth, 2006; Ma et al., 2008). While the lateral line system has led to some remarkable discoveries, it has some potential weaknesses. First, the physical arrangement of neuromast hair cells and supporting cells is quite different from the auditory sensory epithelia in vertebrate inner ears. Secondly, most of the zebrafish lateral line studies are done within the first 5 days following fertilization. Thus, it is unclear whether the observed effects are influenced by developmental plasticity or distinct hair cell death and regenerative pathways. For these reasons, researchers are beginning to perform these studies in the inner ear of adult fishes (Smith et al., 2006; Schuck and Smith, 2009; Uribe et al., 2013b).

### The Teleost Inner Ear

Unlike mammals, fishes have no external or middle ears. Instead, they possess two inner ears located adjacent to the brain enclosed in a pair of capsules in the cranium. The auditory system of teleost fishes consists of three sensory otolithic end organs, the saccule, lagena and utricle (Bever and Fekete, 2002; Nicolson, 2005; Inoue et al., 2013; Figures 1E,F). These end organ compartments are filled with endolymphatic fluid and are interconnected by vestibular semicircular canals (Nicolson, 2005; Cruz et al., 2009). In many ostariophysan fish, like zebrafish, these fluid-filled compartments are attached at their posterior end to a set of four small bones, the Weberian ossicles, which are interconnected by ligaments, and form a connection to the air-filled swim bladder (Bang et al., 2001). The Weberian ossicles function as an accessory hearing structure to transmit sound vibrations from the swim bladder to the inner ear sensory end organs. Fishes do not have a dedicated auditory organ like the mammalian cochlea, and while the otolithic organs have both vestibular and auditory functions it is generally thought that the utricle is primarily a vestibular organ, the saccule is thought to be primarily responsible for sound detection in most fish species, and the lagena supplements the functions of the saccule with roles in both orientation and hearing (Popper et al., 2003; Kwak et al., 2006; Khorevin, 2008). These pouch-like organs contain an area lined with sensory epithelia, referred to as a macula. The macula consists of a basal lamina which underlies a layer of non-sensory supporting cells with interspersed hair cells (Oesterle and Stone, 2008; Inoue et al., 2013). Neurons extend between the supporting cells forming synaptic connections with hair cells (Szabo et al., 2007; Tanimoto et al., 2009). Ciliary bundles consisting of one kinocilium and multiple stereocilia project from the hair cell body into the lumen where they contact an otolith, a calcium carbonate structure found inside the lumen of each end organ (Bever and Fekete, 2002; Nicolson, 2005; Cruz et al., 2009; Inoue et al., 2013). Stereocilia are connected by elastic tip links and when deflected by the denser otolith, transduce either excitatory or inhibitory stimuli depending upon their directional movement (Nicolson, 2005). Newly formed hair cells may arise throughout the teleost inner ear maculae (Popper and Hoxter, 1990; Lombarte and Popper, 1994; Higgs et al., 2001).

### Similarities and Differences Between Teleost and Mammalian Hair Cells

Since fishes are the common ancestor of all tetrapods (Clack, 2012), it is not surprising that teleost and mammalian hair cells share many fundamental features (Coffin et al., 2004). All sensory hair cells are elongate epithelial cells. On their apical surface there is a ciliary bundle composed of multiple actin-rich stereocilia and a single, eccentrically-placed kinocilium (a true cilium with 9 + 2 microtubule pattern; Popper, 1983). The stereocilia are graded in size, with the longest ones lying closest to the kinocilium, and shorter stereocilia positioned in rows in steplike positions away from the kinocilium. Filamentous tip links connect each stereocilia to its nearest neighbor (Pickles et al., 1988). These tip links are composed of two cadherin molecules- protocadherin 15 (PCDH15) and cadherin 23 (CDH23; Siemens et al., 2004; Ahmed et al., 2006), but it is likely that other proteins are also involved. It is hypothesized that the tip links interact with myosin motors that move along actin filaments in the stereocilia (Howard and Hudspeth, 1987; Holt and Corey, 2000; Furness et al., 2010).

Mechanotransduction occurs when hair cell stereociliary bundles are deflected toward the kinocilium, stretching the tip links, opening cation-selective channels, and thus depolarizing the cell, while deflection away from the kinocilium hyperpolarizes the cell (reviewed in Strassmaier and Gillespie, 2002). The precise structure of the mechanotransductive channel is currently not known but may incorporate transmembrane channel-like (TMC) 1 and 2 and TMIE proteins (Pan et al., 2013; Zhao et al., 2014). As this research has only been done using mammalian hair cells, there is no data available on the mechanotransduction channel structures in fishes.

There are obvious morphological differences between the hair cells of the inner ear and lateral line of teleosts and those of the mammalian cochlea. For example, cochlear hair cells are arranged in precise rows while the hair cells of teleost maculae are found as patches of fairly equally-spaced hair cells surrounded by supporting cells, similar to those of the mammalian utricle and saccule (Corwin and Warchol, 1991). Also, while auditory, vestibular, and lateral line teleost hair bundles are generally conical in shape with a kinocilium and many rows of stereocilia, mammalian cochlear hair bundles lose the kinocilium upon maturation and have stereocilia organized in fewer rows (3–4) arranged in a more or less pronounced W-shape (reviewed in Furness and Hackney, 2006).

Another morphological difference between teleost and mammalian hair cells that may represent a structural barrier to hair cell regeneration in mammals is cytoskeletal organization. A comparative study of six vertebrates, including zebrafish and dogfish sharks, showed that mammalian supporting cells have very large circumferential F-actin belts and intercellular junctions that express E-cadherin (Burns et al., 2013). However, supporting cells in zebrafish and adult sharks have thin F-actin belts and zebrafish have little E-cadherin. This suggests that fish supporting cells are able to divide and form new hair cells at least partially due to their unique cytoskeletal and cell adhesion characteristics.

Teleost hair cells exhibit considerable heterogeneity, but there are two basic types: Type I-like and Type II hair cells. Type I-like hair cells are located near the macular striola and have both afferent and efferent innervation (Popper, 2000). They are characterized by large, subnuclear bodies of endoplasmic reticulum, large mitochondria, and smaller synaptic bodies associated with synapses (Chang et al., 1992). Type II hair cells are extrastriolar with mostly afferent innervation. Studies suggest that the lateral line canal neuromasts are probably Type I-like cells and free neuromasts may be Type II hair cells (Song et al., 1995), although these identifications have been questioned (Van Trump et al., 2010; Brown et al., 2011). The mammalian cochlea also has two distinct hair cell types. There are inner hair cells (IHCs), the primary auditory receptors, which send afferent signals to the auditory processing centers of the brain, and outer hair cells (OHCs), which receive efferent signals from the brain and act as a cochlear amplifier which sharpens the frequency tuning curves along the length of the cochlea (Vater et al., 2004). It is thought that IHCs and OHCs are homologous to Type I-like and Type II hair cells, respectively. Mammalian vestibular Type I hair cells are amphora or cylindrically shaped but with a constricted neck and Type II hair cells are cylindrically shaped (Kevetter and Correia, 1996). A large nerve calyx surrounds the mammalian IHC hair cell, while this is usually not found in teleost hair cells (Popper, 2000).

Recently, whole-cell patch clamp analysis of zebrafish hair cells has allowed comparison of the physiological properties between hair cells from different locations (lateral line vs. inner ear) and with mammals (Olt et al., 2014). This study showed that all zebrafish hair cells exhibit a delayed rectifier K^+^ current, but mature lateral line hair cells located in the center of neuromasts have large A-type K^+^ currents (*I*_A_) while immature hair cells at the neuromast edge expressed a large conductance Ca^2 +^ -activated K^+^ current (*I*_K, Ca_) and a small *I*_A_. Further, mature zebrafish hair cells physiologically resembled those of other lower vertebrates, and to some extent, the hair cells from immature mammalian vestibular and auditory systems (Olt et al., 2014). However, it is not certain whether zebrafish hair cell physiology resembles that of clinically relevant mature mammalian hair cells.

## Causes and Mechanisms of Hair Cell Damage

### Ototoxins

Fish inner ear and lateral line hair cells may be damaged by many of the same ototoxic chemicals that cause impaired auditory and vestibular function in mammals. These substances include heavy metals, platinum-based drugs, aminoglycoside antibiotics and alkaloids (Yan et al., 1991; Lombarte et al., 1993; Song et al., 1995; Harris et al., 2003; Ton and Parng, 2005; Hernández et al., 2006, 2007; Santos et al., 2006; Chiu et al., 2008; Ma et al., 2008; Olivari et al., 2008; Van Trump et al., 2010; Buck et al., 2012). Fish species tested with ototoxins include goldfish (*Carassius auratus*, Ramcharitar and Brack, 2010), Atlantic cod (*Gadus morhua*, Faucher et al., 2009), Mexican blind cavefish (*Astyanax mexicanus*, Van Trump et al., 2010), oscar (*Astronotus ocellatus*, Lombarte et al., 1993; Song et al., 1995), and zebrafish (*Danio rerio*, Hernández et al., 2006, 2007; Santos et al., 2006; Olivari et al., 2008; Van Trump et al., 2010; Uribe et al., 2013a). Ototoxic exposure of the lateral line can be accomplished by dissolving chemicals in the growth media of fish larvae (Harris et al., 2003; Ton and Parng, 2005; Hernández et al., 2006, 2007; Santos et al., 2006; Ma et al., 2008; Olivari et al., 2008; Van Trump et al., 2010), while exposure of the inner ear can be done via systemic or inner ear injection (Yan et al., 1991; Lombarte et al., 1993; Faucher et al., 2009; Uribe et al., 2013b). The degree of hair cell damage depends on ototoxin concentration (Yan et al., 1991; Ton and Parng, 2005; Hernández et al., 2006; Olivari et al., 2008) and exposure time (Song et al., 1995).

One heavy metal that is ototoxic to fish hair cells is copper. Treatment with 1 μM copper sulfate for 2 h did not cause morphological damage to larval zebrafish neuromasts, but hair cells were completely damaged at 50 μM (Hernández et al., 2006). Copper can also damage supporting cells. At 10 μM exposure, mantle and interneuromastic cells are moderately damaged, while at 50 μM, mantle, but not interneuromastic cells, are completely destroyed (Hernández et al., 2007). Mammalian studies have shown that copper transporters and pumps are responsible for cisplatin influx, sequestration and efflux (Ding et al., 2011). However, copper sulfate, an inhibitor of the copper transporter, CTR1, which modulates cisplatin influx in rodent models, can cause ototoxicity or otoprotection against cisplatin depending on dosage (More et al., 2010; Ding et al., 2011). In fact, CTR1 mRNA is expressed in zebrafish lateral line hair cells (McDermott et al., 2007), although, the level of CTR1 protein expression and its functionality has not yet been studied in either zebrafish lateral line or inner ear hair cells. Further, mRNA tissue expression of copper transporter genes can vary significantly between mammals, zebrafish and other fish species after copper exposure (Leung et al., 2014). Therefore, mammalian hair cells may respond differently than fish to copper-based compounds.

**Figure 2:**
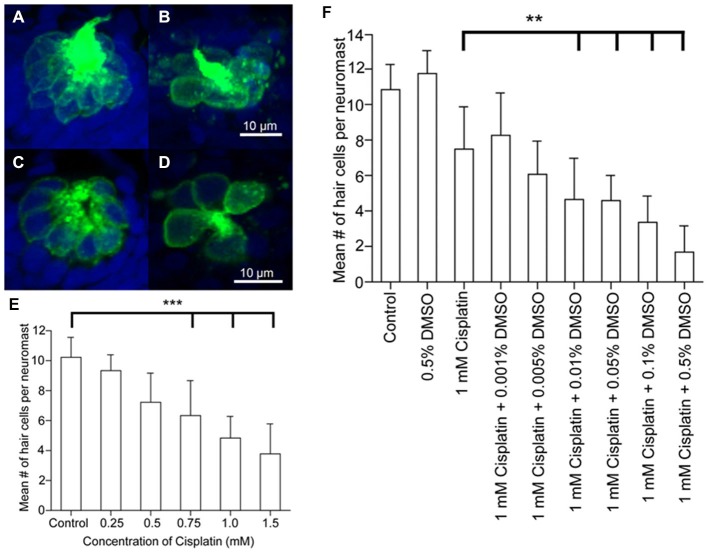
**Dose response curves following cisplatin and DMSO treatment**. Brn3c-green fluorescent protein (GFP) transgenic zebrafish were exposed to varying doses of cisplatin for 4 h, then fixed and co-labeled with TO-PRO-3 (blue), and the GFP-tagged hair cells (green). Neuromasts were imaged using confocal microscopy. **(A,B)** *Z*-stack projections of two neuromasts under different treatment conditions showing the entire neuromast structure. **(C,D)** Slices from the same neuromasts as in A, B demonstrating the membrane-bound GFP label surrounding the nuclear dye. Neuromasts of untreated controls **(A,C)** and 1 mM cisplatin-treated fish **(B,D). (E)** Dose-response curve for the effect of cisplatin on neuromast hair cell number. ***p < 0.001 when individual treatments are compared to untreated controls. **(F)** Dose-response curve showing the synergistic effects of cisplatin and DMSO on neuromast hair cell number. **p < 0.01 when individual treatments are compared to untreated controls (modified from Uribe et al., 2013a).

Zebrafish hair cells can also be damaged by the anticancer platinum-based compound, cisplatin, which is also ototoxic in mammals (Ton and Parng, 2005; Ou et al., 2007; Chiu et al., 2008; Owens et al., 2008; Giari et al., 2012). Transmission and scanning electron microscopy shows that cisplatin damages inner ear more than lateral line hair cells with greater numbers of damaged mitochondria in the inner ear cells than in the lateral line (Giari et al., 2012). The mechanism responsible for increased inner ear sensitivity is not known, although lateral line hair cells require mechanotransduction for cisplatin influx (Thomas et al., 2013). However, rodent studies suggest that cisplatin uptake in mammals may depend upon membranous transporters (Ciarimboli et al., 2010; More et al., 2010; Ding et al., 2011; Ciarimboli, 2012). It is unknown whether cisplatin uptake in zebrafish inner ear hair cells is mechanotransduction or transporter dependent. Cisplatin-mediated hair cell damage may be modulated by other chemicals. For example, ototoxicity increases in zebrafish lateral line hair cells treated with cisplatin when they are also exposed to the pharmaceutical solvent, DMSO, although DMSO treatment alone does not cause hair cell death (Uribe et al., 2013a; Figure 2). Cisplatin and other anti-cancer drugs can produce synergistic ototoxic effects in the zebrafish lateral line (Hirose et al., 2011). Experiments investigating DMSO and synergistic effects have not been performed in zebrafish inner ear hair cells, however, in rats, DMSO can cause hair cell death by activating apoptotic pathways (Qi et al., 2008). Studies in guinea pigs have also found that DMSO has an otoprotective effect which can be enhanced by co-application of a synergistic agent (Momin et al., 2011). These results suggest that platinum-based compounds and synergistic agents may cause different ototoxic effects in mammalian vs. zebrafish hair cell models.

Aminoglycoside antibiotics that are ototoxic in mammals can also cause hair cell death in fish (Ton and Parng, 2005; Chiu et al., 2008). For example, gentamicin and neomycin cause ototoxicity in the zebrafish lateral line (Ton and Parng, 2005), and streptomycin damages the superficial and canal neuromasts of goldfish (Higgs and Radford, 2013). Although different levels of gentamicin-induced damage in superficial vs. canal neuromasts have been reported (Song et al., 1995), another study showed that zebrafish superficial and canal neuromasts were damaged to a similar extent when exposed to gentamicin (Van Trump et al., 2010). Therefore, results obtained with aminoglycosides may be species specific and warrant careful consideration regarding choice of a particular fish model. Zebrafish inner ear studies show that gentamicin injection also damages hair cells in the saccular and utricular sensory epithelium and causes auditory functional deficits (Uribe et al., 2013b).

Rodent models of aminoglycoside ototoxicity can present disadvantages. Induction of aminoglycoside-mediated ototoxicity in mice often requires drug treatments that cause significant mortality and complex delivery methods (Murillo-Cuesta et al., 2010). Furthermore, gentamicin studies in guinea pigs demonstrate that this drug is more vestibulotoxic than ototoxic (Zhai et al., 2010). Aminoglycoside studies in mice have also exhibited distributed hair cell damage patterns where outer hair cells are mostly destroyed but many inner hair cells are left intact (Taylor et al., 2008). Thus, the ototoxic effects of aminoglycosides on fish models may be different than that of their mammalian counterparts.

Developmental factors may play a significant and complicating role in zebrafish models of aminoglycoside ototoxicity. For example, in larval lateral line studies, hair cell susceptibility to neomycin increases during later stages of development (Murakami et al., 2003; Santos et al., 2006). Specifically, zebrafish treated four days post-fertilization exhibit little hair cell damage while older fish have many more damaged hair cells. This is generally the opposite of mammalian organisms where greater sensitivity to ototoxins is observed during early developmental stages and greater resistance is found in adult specimens (Henley and Rybak, 1995). Further, maturation-related sensitivity in the zebrafish lateral line has been associated with hair cell type as immature Type I-like hair cells are less susceptible to neomycin but are more strongly affected as they approach maturity (Harris et al., 2003). No studies to date have studied the role of developmental drug sensitivity in fish inner ear hair cells. Therefore, studies of aminoglycosides, and potentially other ototoxic drugs in fish models, should carefully consider how development might affect experimental outcomes.

Transgenic zebrafish expressing fluorescent protein reporters can exhibit impaired hearing. Zebrafish expressing green fluorescent protein (GFP) under the control of the *brn3c* promoter *Tg(Brn3c:GFP)* have elevated hearing threshold shifts compared to wild-type controls (Uribe et al., 2013b). This is similar to transgenic mouse models where GFP expression in hair cells is correlated with hearing deficits (Wenzel et al., 2007), while lower levels of GFP in these cells causes no hearing loss (Wang et al., 2013a). It is not certain whether GFP acts as an ototoxin. However, long-term GFP expression in transgenic mice has been linked to aberrant physiology (Huang et al., 2000). Future work will be required to determine whether the expression of fluorescent reporters causes ototoxic effects in zebrafish as well as in mammalian models.

### Acoustic Damage

**Figure 3:**
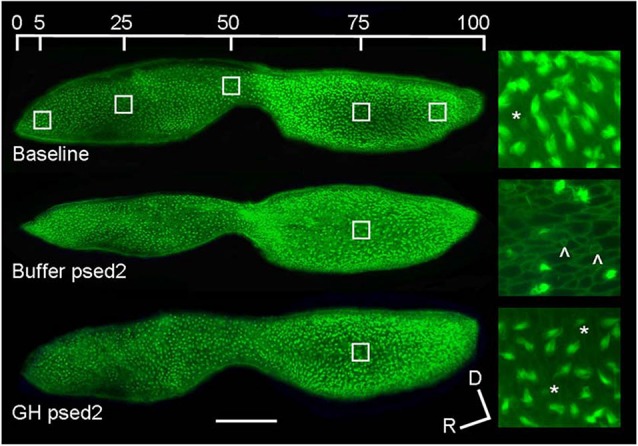
**Effect of acoustic exposure on zebrafish saccular hair cell bundle density**. Phalloidin-labeled saccular epithelia of baseline, buffer-injected, and GH-injected zebrafish at post-sound exposure day 2 (psed2). The upper image shows the five locations of hair cell counts along the rostral-caudal axis of the saccule. The enlarged images to the right of the saccules are representative 100X images of saccules at 75% along the rostral-caudal axis. Scale bar, 100 μm, D = dorsal, R = rostral, * = presumed newly formed hair cell bundles, ˆ= scar formation characteristic of hair cell loss (modified from Sun et al., 2011).

The hair cells of fishes, like mammals, can be damaged by a variety of sound stimuli. For example, 48 h of white noise at 180 dB re: 1 μPa produces hair cell damage in the lagena and saccule of goldfish with saccular damage being particularly localized in the central and caudal regions (Smith et al., 2006). Hair cell loss was also correlated with increased auditory threshold shifts over a range of frequencies (0.2–2 kHz). The goldfish saccule is tonotopically organized, and hair cells in discrete saccular locations are susceptible to stimuli of different frequencies, with low and high frequency sounds damaging hair cells of the caudal and rostral portions of the saccule, respectively (Smith et al., 2011). Similarly, low frequency sound exposure in zebrafish causes distributed hair cell damage in the caudal portion of the saccule (Schuck and Smith, 2009; Sun et al., 2011; Figure 3). Distributed damage patterns in the rostral and caudal portions of the saccule have also been identified in hybrid striped bass (white bass, *Morone chrysops* × striped bass, *Morone saxatilis*) and tilapia (*Oreochromis mossambicus*) exposed to pile driver noise (Casper et al., 2013). Similarly, studies conducted in mammalian models show that acoustic damage is often concentrated in different areas of the cochlea with lower frequencies typically damaging mainly outer hair cells and higher frequencies are required to damage inner hair cells (Stockwell et al., 1969; Lim, 1976; Park et al., 2013). As in fish, the mammalian auditory epithelia is tonotopically organized, although, mammalian organization is much more sophisticated (Mann and Kelley, 2011). However, it is unknown whether fish auditory sensory epithelia undergo tonotopic reorganization in response to acoustic damage, which in mammalian models includes changes in electrophysiological response, e.g., threshold response shifts and altered frequency detection, in different regions of the sensory epithelia (Wang et al., 2013b). In general, the cellular damage exhibited by fish and mammals after acoustic over-stimulation is similar. Immediately after zebrafish are sound exposed, hair cell stereociliary numbers are reduced, there are shorter and thinner ciliary bundles, and epithelial lesions where hair cells have been removed (Schuck and Smith, 2009). In mammalian models, after noise exposure, stereocilia are disarrayed or collapsed, and there is scarring where hair cells were located (Stockwell et al., 1969; Lim, 1976; Wang et al., 2002). Thus, fish and mammalian hair cells may be affected similarly by damaging noise stimuli, but there is still limited data on the effects of acoustic trauma on teleost hair cells.

### Hair Cell Damage Mechanisms

Researchers have begun to characterize the mechanisms involved in fish hair cell death and how they relate to mammalian models. The terminal deoxynucleotidyl transferase dUTP nick end labeling (TUNEL) assay shows that noise exposure and ototoxins modulate apoptosis in zebrafish lateral line and inner ear hair cells (Sun et al., 2011; Chang et al., 2013; Hong et al., 2013; Song et al., 2013; Uribe et al., 2013b). Various indicators of apoptosis, e.g., nuclear condensation and fragmentation, can appear rapidly (30 min) in larval zebrafish neuromasts after cisplatin treatment (Ou et al., 2007). These results are consistent with noise-induced damage and ototoxicity studies conducted using TUNEL in mammalian organisms (Ahn et al., 2005; Chung et al., 2007; Taylor et al., 2008; Fu et al., 2012).

Non-apoptotic mechanisms can also be activated during fish hair cell death. For example, administration of low concentrations of copper causes apoptosis in zebrafish lateral line hair cells but higher concentrations modulate both apoptotic and necrotic mechanisms (Olivari et al., 2008). Non-apoptotic pathways are also activated in mammalian hair cell death. Mice exposed to the aminoglycoside, kanamycin, undergo hair cell death without activation of markers integral to the apoptotic pathway (Jiang et al., 2006). Further, hair cell death in noise-exposed mice can be due to activation of both apoptotic and necrotic pathways (Zheng et al., 2014).

Different pathways in fish hair cells may be targeted by specific ototoxins. Mutational studies show that multiple genes modulate aminoglycoside susceptibility in the larval zebrafish lateral line (Owens et al., 2008). It is likely that these genes function in different pathways, because they provide differential resistance to neomycin. Also, in zebrafish larvae, neomycin and gentamicin ototoxicity follows different time courses suggesting that different cell death mechanisms are targeted (Owens et al., 2009). Screens in larval zebrafish using signal transduction inhibitors targeting the molecules, Bax, Bcl2 and p53, suggest that specific pathways are associated with different otoprotective effects against various aminoglycosides and cisplatin (Coffin et al., 2013a). Studies conducted in mouse models also suggest that different ototoxins can signal through different hair cell death pathways (Jiang et al., 2006; Zheng et al., 2014). However, no studies have yet compared whether specific ototoxic drugs modulate the same apoptotic or necrotic pathways in zebrafish or mammalian models. Recent studies suggest that zebrafish auditory cell apoptosis and necrosis may signal through markers found in rodent models, e.g., Bax, Bcl2, p38, p53 and cytochrome c (Coffin et al., 2013a,b; Shin et al., 2014).

The role of mechanotransducive mechanisms in hair cell death is a potential point of divergence between fish and mammals. A study in the zebrafish lateral line shows that mechanotransduction is required to modulate the ototoxic effects of cisplatin (Thomas et al., 2013). The alkaloid, quinine, an ototoxic mechanotransduction blocker in mammals (Alharazneh et al., 2011), reduces mechanotransduction in the zebrafish lateral line and prevented cisplatin uptake (Thomas et al., 2013). However, work conducted with rodent models indicates that cisplatin uptake in mammals depends upon a variety of membranous transporters, e.g., CTR1, and, therefore, may not require mechanotransduction (Ciarimboli et al., 2010; More et al., 2010; Ding et al., 2011; Ciarimboli, 2012). These findings suggest that cisplatin influx in fish proceeds through a different mechanism than in mammals. An additional complicating factor is that hair cell development may intersect with mechanotransduction-modulated ototoxicity and affect the susceptibility of zebrafish to aminoglycosides. As zebrafish hair cells age they become more susceptible to neomycin, and age-related effects are not dependent upon the onset of functional mechanotransduction (Santos et al., 2006). Much additional work is needed before conclusions can be drawn as to whether zebrafish hair cell death mechanisms are largely equivalent to their mammalian counterparts and if the same pathways are involved in the response to specific ototoxins.

## Hair Cell Regeneration

### Cellular Differentiation and Proliferation

Hair cells can regenerate after damage through transdifferentiation or proliferation of supporting cells (Corwin and Oberholtzer, 1997; Warchol, 2010; Burns and Corwin, 2013; Rubel et al., 2013). Studies conducted using tritium and bromodeoxyuridine (BrdU) labeling show that hair cells in the teleost lateral line and inner ear can undergo continuous proliferation (Jørgensen, 1991; Lanford et al., 1996; Sun et al., 2011). Following acoustic trauma, fish can regenerate hair cells to control levels within one to two weeks, and functional recovery of hearing results (Smith et al., 2006; Faucher et al., 2009). However, regeneration may not proceed, at least in the lateral line, if a certain level of damage is reached. The regenerative potential of neuromasts can be dose-dependent and as damage increases, other cell types from which new hair cells are derived may be permanently destroyed (Hernández et al., 2006).

Several studies have shown that hair cells can regenerate from mitotic and proliferating supporting cells. In the oscar and goldfish inner ear saccule, supporting cells can enter mitotic S-phase and become hair cell precursors (Presson et al., 1995, 1996). In the zebrafish lateral line, hair cells normally undergo programmed cell death during development but are restored from mantle supporting cells at the periphery of the neuromast after S-phase has occurred (Williams and Holder, 2000; Harris et al., 2003). Proliferating supporting cells may either stay in the periphery or migrate inwards and their numbers increase after drug-induced hair cell death. Another zebrafish lateral line study found that most new hair cells are from proliferating supporting cells and that there are two sets of these cells within neuromasts (Ma et al., 2008). One group functions as the progenitors of hair cells and is centrally located. The other is peripheral and of uncertain function. This suggests that there may be functional specializations among neuromast supporting cell populations. These findings are similar to those of transgenic neonatal mouse models where new hairs cells can also proliferate from supporting cells (Shi et al., 2013). In contrast, hair cell regeneration does not occur in adult guinea pigs following damage to both hair and supporting cells suggesting that supporting cells are critical for regeneration (Izumikawa et al., 2008).

Fish hair cells can also regenerate from transdifferentiation of supporting cells. Inflicting high levels of damage to neuromasts causes replacement of hair cells from dividing surrounding supporting cells (Hernández et al., 2007). However, lower levels of damage can cause non-dividing precursor cells to differentiate into hair cells. In developing zebrafish embryos, laser-ablated inner ear hair cells were replaced by supporting cells that underwent transdifferentiation into new hair cells without mitotic proliferation (Millimaki et al., 2010). Transdifferentiation of supporting cells has also been observed in genetically-manipulated mammalian models (Izumikawa et al., 2005).

One potential complication involved in studying cellular differentiation and proliferation in zebrafish is that their lateral line and inner ear hair cells are continually replaced during life (Popper and Hoxter, 1984; Lombarte and Popper, 1994; Higgs et al., 2001, 2003). This could make it difficult to discriminate hair cell regeneration stemming from transdifferentiation as opposed to supporting cell division. However, the application of new technologies, such as laser-scanning confocal microscopy coupled with selective plane illumination microscopy, could facilitate study of these processes by allowing continuous videomicroscopy of different cell types labeled with unique fluorescent markers (Pinto-Teixeira et al., 2013). Thus, this technique could allow investigators to determine which cell types are involved in regeneration and whether new hair cells are produced by transdifferentiation or supporting cell division.

**Figure 4:**
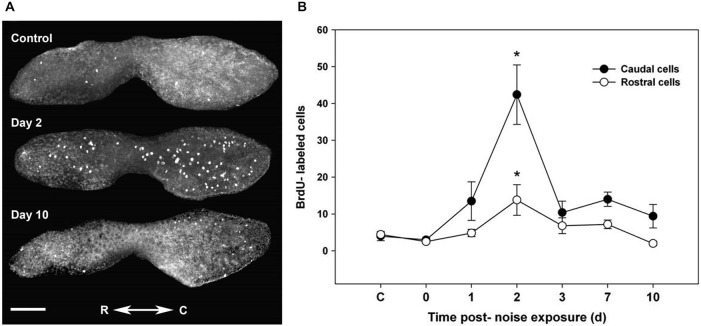
**Proliferation follows acoustic trauma to the zebrafish inner ear. (A)** BrdU-labeled proliferating cells in a control zebrafish saccule and in saccules dissected for 2 and 10 days post-noise exposure. C = caudal, R = rostral; scale bar = 100 μm. **(B)** Mean (±SE) BrdU-labeled zebrafish saccular cells as a function of time following sound exposure (*p < 0.001; *n* = 6). Adapted from Hearing Research, Vol. 253, J.B. Schuck, M.E. Smith, Cell proliferation follows acoustically-induced hair cell bundle loss in the zebrafish saccule, 2009, with permission from Elsevier.

Hair cells in the fish inner ear may exhibit differential spatial patterns of regeneration. In noise-exposed goldfish, hair cell bundle density recovered in the central but not in the caudal portion of the saccule during the study time interval (Smith et al., 2006). After acoustic exposure, hair cells largely regenerated in the caudal region of the saccule, where higher hair cell loss and subsequent mitotic activity was greater than that found in the rostral region (Schuck and Smith, 2009; Figure 4). This was a result of the low frequency sound stimuli used and the tonotopic organization of the teleost saccule (Smith et al., 2011).

### Regenerative Mechanisms

**Figure 5:**
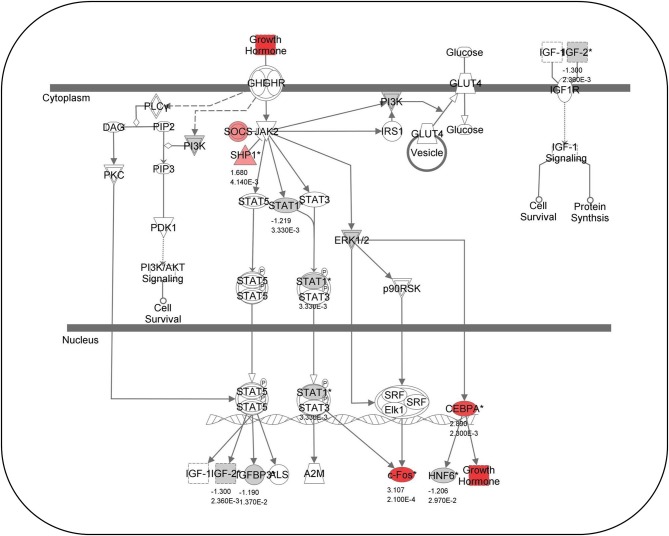
**Growth hormone-related gene expression**. Canonical pathway analysis showing known growth-hormone related pathways. Red colored genes are up-regulated and gray-colored genes are down-regulated 2 days following acoustical trauma. Numbers below genes represent fold changes and *P*-values (Modified from Schuck et al., 2011).

The ability of fish hair cells to regenerate has provoked great interest in identifying the genes and molecular mechanisms that control their differentiation and proliferation. Recent studies have used microarrays and next generation sequencing to identify genes that are activated after hair cell destruction. A microarray performed on zebrafish exposed to noise showed that many gene transcripts were either up- or down-regulated in the inner ear (Schuck et al., 2011; Figure 5). Growth hormone is particularly upregulated while myosin light chain, myosin heavy chain, and major histocompatibility complex I genes were significantly down-regulated. A subsequent study demonstrated that growth hormone can promote regeneration of acoustically- damaged inner ear hair cells in the saccule, utricle and lagena (Sun et al., 2011). Studies in mammalian models suggest that hormones and growth factors can modulate hair cell regeneration. For example, transforming growth factor α and insulin supplemented with epidermal growth factor induced cellular regeneration in mouse vestibular sensory epithelia (Yamashita and Oesterle, 1995). Although, the action of some growth factors, e.g., insulin-like growth factor-1, in mammals may be confined to protective and not regenerative effects (Yamamoto et al., 2014).

Digital gene expression, a form of next generation sequencing, has also been used to identify genes that modulate hair cell regeneration in adult and larval zebrafish (Liang et al., 2012). This showed that the *stat3*/*socs3* pathway can modulate hair cell production in the lateral line during development and the adult inner ear during hair cell regeneration, and its expression and activity is associated with supporting cells, differentiating hair cells, and cell division. In mouse models, upstream effectors of Stat3 may be involved in hair cell survival (Hertzano et al., 2004), but no role for Stat3 in hair cell regeneration has yet been reported in mammals.

While microarrays and digital gene expression studies typically use tissue preparations, gene expression can also be studied by collecting samples from individual progenitor cells. For example, lateral line mantle cells can be fluorescently-labeled, isolated by flow cytometry and then subjected to whole-transcriptome microarray analysis (Steiner et al., 2014). This approach revealed that after hair cell ablation, transcripts coding for transmembrane receptors and cell-adhesion molecules increased. Some of these transcripts were confined to particular mantle cell subsets in specific neuromast regions. Further, transcriptional levels followed a temporal course with maximal changes occurring within 3–5 h following ablation. Using this methodology, it may possible to further elaborate the mechanistic differences between mammalian and non-mammalian hair cell progenitors.

Chemical screens have also been used to identify compounds that enhance or inhibit hair cell regeneration in the zebrafish lateral line. For example, a chemical screen has been used to identify synthetic glucocorticoid enhancers that promote hair cell regeneration by increasing mitotic activity (Namdaran et al., 2012). This study also identified inhibitors that either reduced hair cell regeneration or prevented supporting cell proliferation, and some of these drugs targeted cell division mechanisms.

Zebrafish studies show that Wnt signaling is involved in hair cell regeneration. In neuromasts, inhibition of Wnt/β-catenin signaling reduces proliferation and hair cell differentiation while activation of Wnt increases hair cell numbers and promotes supporting cells to reenter the cell cycle and proliferate (Head et al., 2013; Jacques et al., 2013). Further, Wnt/β-catenin activation causes increased hair cell regeneration (Jacques et al., 2013). Neuromast size is also regulated by a negative feedback loop that incorporates Wnt signaling activity (Wada et al., 2013). This promotes surrounding cells to proliferate and is inhibited by Dkk activity from differentiated sensory cells. An analysis of RNA transcripts expressed in the zebrafish lateral line following neomycin-induced damage showed that Wnt/β-catenin signaling is down-regulated during earlier time points but becomes up-regulated later (Jiang et al., 2014). This suggests that Wnt is necessary for hair cell proliferation but not immediately after hair cell damage.

Studies of Wnt signaling in zebrafish indicate that the transcription factor Sox2 is involved in hair cell proliferation and transdifferentiation. In one study, new hair cells were derived from proliferating Sox2-positive cells in the prosensory domain (Jacques et al., 2013), and Sox2 is highly expressed in most proliferating neuromast progenitor cells (Hernández et al., 2007). Sox2 is also required for transdifferentiation of supporting cells (Millimaki et al., 2010). Another important regulator of Wnt/β-catenin signaling is ErbB/Neuregulin, which can act upstream to regulate the proliferation of zebrafish lateral line interneuromast cells and the development of intercalary neuromasts (Lush and Piotrowski, 2014b). ErbB signaling is required for the migration of Schwann cells and in the absence of ErbB signaling or Schwann cells, Wnt/β-catenin and Fgf (fibroblast growth factor) signaling is increased. Further, Wnt/β-catenin signaling is required for interneuromast proliferation, while protoneuromast formation and cellular differentiation requires Fgf signaling (Lush and Piotrowski, 2014b). When ErbB/Neuregulin signaling is reduced, Notch signaling is induced many hours after activation of Wnt/β-catenin but before Fgf signaling begins.

The Notch signaling pathway is also a key modulator of hair cell regeneration in the zebrafish lateral line. When supporting cell proliferation and formation of hair cell progenitors is maximal, transcript levels of the Notch pathway components, *notch3, deltaA*, and *atoh1a*, increase (Ma et al., 2008). However, inhibiting Notch signaling can also cause hair cell regeneration (Moon et al., 2011). These studies suggest that the Notch pathway modulates regeneration as part of a complex interplay of signaling elements. In the zebrafish *mind bomb* mutant, Delta-Notch signaling was responsible for controlling supporting cell differentiation into inner ear hair cells (Haddon et al., 1998). A time course analysis of lateral line hair cell regeneration in zebrafish found that Notch signaling is inhibited immediately following hair cell damage (Jiang et al., 2014). The Notch target genes, *her4.1, notch3*, and *sox2*, were down-regulated at 1 h, but were up-regulated between 3 and 5 h after damage. *Atoh1a*, a Notch target gene, was up-regulated at 1 h and *deltaD*, a target of Atoh1a, was up-regulated between 3 and 5 h following damage. The inhibition of Notch, and the up-regulation of *atoh1a* and *deltaD*, suggests that during hair cell regeneration, Notch inhibition needs to be relieved so that supporting cells can leave a quiescent state and begin proliferation. Constitutively expressing Notch can prevent hair cell proliferation from supporting cell progenitors, while eliminating Notch activity produces increased numbers of hair cell progenitors and ectopic hair cells (Wibowo et al., 2011). Further, Notch3 activity prevents supporting cells from becoming hair cell progenitors outside of the polar compartments where hair cells regenerate and establish directional symmetry. Thus, when Notch activity is low and Atoh1a expression is higher within the polar compartment, hair cell progenitor division and immature hair cell formation increases.

Differentiation of hair cell progenitors requires expression of the transcription factor, Atoh1 (Ma and Raible, 2009; Groves et al., 2013). Gain and loss-of-function techniques have been used to characterize the role of Atoh1 in hair cell regeneration. Knockdown of Atoh1a reduced hair cell formation in the zebrafish inner ear, while rescuing expression restored hair cells (Millimaki et al., 2007). However, excess Atoh1a expression did not produce ectopic or excess hair cells, as found in rodent models (Zheng and Gao, 2000). Interestingly, adjacent to the lateral line, excess Atoh1a increased ectopic hair cell placement (Millimaki et al., 2007). These results suggest that Atoh1 signaling may modulate inner ear and lateral line hair cell regeneration differently and may promote different effects in zebrafish and mammalian models. Knockdown of Atoh1 can also reduce zebrafish neuromast development; whereas, rescue or over-expression induces the activity of NeuroD, which is required for hair cell differentiation (Sarrazin et al., 2006). However, mantle and interneuromastic cells are not affected by reduction of Atoh1 protein. Activating Atoh1a signaling in the zebrafish inner ear at different time intervals can cause hair cell formation in distinct spatial regions (Sweet et al., 2011). For example, activation at 18 h post fertilization (hpf) causes formation of ectopic hair cells in the region normally occupied by the utricular and saccular maculae. At 36 hpf, activation caused two discrete and enlarged maculae to appear with an intervening region devoid of hair cells, while at 48 hpf, few hair cells develop and no ectopic cells were observed. Although increasing Atoh1 expression in mammalian models can cause hair cell regeneration, these cells typically exhibit aberrant placement and morphology (Sweet et al., 2011). Thus, future research is required to determine whether results obtained with zebrafish models can be applied to mammalian organisms.

Atoh1a can also regulate hair cell regeneration by modulating ion transport (Go et al., 2010). Specifically, knockdown and inhibition of the plasma membrane calcium transporter ATPase, Atp2b1a (Pmca1), which is controlled by Atoh1a, can affect hair cell regeneration. Knockdown of Atp2b1a blocks calcium export and increases hair cell progenitors. Thus, Atp2b1a might, under control of Atoh1a, affect progenitor cell proliferation and hair cell differentiation via a calcium signaling mechanism. Another ATPase gene, *atp1b2b*, which regulates Na^+^/K^+^ transport, can affect hair cell regeneration (Wang et al., 2008). Knockdown of Eya4, a transcriptional co-activator of Atp1b2b, decreased the number of hair cells in the zebrafish inner ear compartment and lateral line during development. Atp1b2b has a similar spatial and temporal expression as Eya4 and is reduced during Eya4 knockdown. Specific knockdown of Atp1b2b also reduced hair cell numbers in the inner ear compartment and lateral line while over-expression restored the normal phenotype, suggesting that Eya4 may regulate hair cell regeneration by modulating the Na^+^/K^+^-ATPase transporter.

As in zebrafish, Wnt, Notch and related signaling is involved in mammalian hair cell proliferation and transdifferentiation during development. For example, inhibition of Notch signaling causes proliferation of supporting cells in mouse cochlea by acting through the Wnt signaling pathway, however, transdifferentiation was Wnt-independent (Li et al., 2015). In the mouse utricle, inhibition of Notch signaling caused transdifferentiation of supporting cells with little mitotic proliferation (Lin et al., 2011). Transdifferentiation of supporting cells is also promoted by over-expressing Atoh1 in the guinea pig inner ear (Izumikawa et al., 2005). Over-expression in supporting cells of the downstream Wnt signaling molecule, β-catenin, caused proliferation in some cells and reduced expression of p27, a cell cycle inhibitor (Shi et al., 2013). Similarly, reducing p27 expression in mouse cochlea causes proliferation of hair and supporting cells (Chen and Segil, 1999; Ono et al., 2009). Interestingly, functional assays show no differences in hearing ability between wild type and p27 mutant animals (Walters et al., 2014). In the zebrafish lateral line, when histone deacetylases are inhibited, the numbers of hair and supporting cells decrease, neuromast cellular proliferation is reduced, and p27 mRNA levels increase (He et al., 2013). These studies suggest that p27 may regulate cellular proliferation in both zebrafish and mammalian models.

Caspase signaling may also play a role in hair cell regeneration. For example, caspase inhibition reduced hair cell death and supporting cell proliferation in zebrafish peripheral neuromasts (Williams and Holder, 2000). This suggests that caspases could be an integral part of a feedback mechanism that controls supporting cell division and entry into mitosis in response to hair cell death. Caspase activity in zebrafish may function similarly to mammalian models where caspases can modulate apoptosis (Zheng et al., 2014). However, hair cells could be continually renewed from supporting cells in the absence of cell death signaling. A study of hair cell regeneration in the oscar saccule and chicken utricle suggests that supporting cells may not require a cell death signal in the oscar in order to proliferate (Wilkins et al., 1999). In an avian study, caspase inhibition caused reduction of hair cell death and supporting cell proliferation (Matsui et al., 2002) similar to findings in the zebrafish lateral line (Williams and Holder, 2000). Teleosts could have a population of supporting cells that undergo continual renewal without a cell death signal requirement or require such a signal for some or all supporting cells. Alternatively, new hair cells might trigger the death of older cells. Further, as the oscar saccule continues to enlarge during adult life, developmental mechanisms may also be implicated in hair cell regeneration (Wilkins et al., 1999). Future research is needed to characterize the roles played by developmental and intracellular cell death signaling mechanisms in teleosts and their effects on hair cell regeneration.

Other mechanisms have been identified that can modulate teleost hair cell regeneration. The phoenix gene (*pho*), which is strongly expressed in supporting cells, affects zebrafish hair cell proliferation and regeneration (Behra et al., 2009). Pho mutants exhibit normal neuromast and hair cell development. However, when progenitor cells are destroyed in the mutants, supporting cell proliferation and hair cell regeneration is greatly reduced. This suggests that Pho may regulate entry into mitosis. Another mechanism involves histone methylation by lysine specific demethylase 1 (LSD1), which can regulate hair and supporting cell proliferation during development (He et al., 2013). Inhibition of LSD1 reduces the number of hair and supporting cells and suppressed neuromast cellular proliferation. Increased inhibition caused apoptosis within neuromasts. While investigating the origin of new hair cells in the zebrafish lateral line, Lin et al. (2013), found that Rb (retinoblastoma protein) and Raf-1 kinase were involved in asymmetric cell division in neuromasts. Blocking the interaction between Rb and Raf-1, and Rb phosphorylation, prevented hair cell regeneration from supporting cells. In a rat model, inhibition of phosphorylated Rb promoted proliferation of supporting cells which transdifferentiated into hair cells by activating the sonic hedgehog pathway (Lu et al., 2013).

While most of the research on hair cell regeneration has focused on protein expression at the gene level, microRNAs (miRNAs), which are small, non-coding RNAs that regulate gene expression by translation repression or mRNA destabilization (Friedman et al., 2009), have been shown to play an important role in hair cell development. For example, miRNAs can regulate hair cell proliferation in embryonic zebrafish inner ears (Wienholds et al., 2005; Li et al., 2010). Knockdown of miR-96, -182 and -183 causes a reduction in the numbers of inner ear hair cells. Conversely, over-expressing miR-96 and -182 promotes hair cell production. These miRNAs are also expressed in the mouse inner ear (Weston et al., 2006), and they have been shown to have roles in inner ear development (Sacheli et al., 2009; Soukup, 2009). Further, miR-96 can regulate differentiation of cochlear hair cells (Kuhn et al., 2011). A more in depth review of miRNAs regulated in vertebrate hair cells is provided elsewhere (Smith and Rajadinakaran, 2013).

## Future Directions

### Otoprotectants

One strategy for preventing damage to hair cells is to administer otoprotective drugs that counteract cellular injury from otherwise beneficial drugs (Coffin et al., 2010; Ou et al., 2010; Esterberg et al., 2013). Otoprotectants mainly work by interfering with antibiotic uptake (Hailey et al., 2012; Ou et al., 2012) or inhibiting generation of reactive oxygen species (Le Prell et al., 2007; Wu et al., 2014). The antioxidant epicatechin protects both mammalian and zebrafish hair cells against cisplatin damage and decreases reactive oxygen species generation (Kim et al., 2008). Similarly, Shin et al. (2013), demonstrated that the drug 3-amino-3-(4-fluoro-phenyl)-1H-quinoline-2, 4-dione (KR-22332) provided otoprotective benefits against cisplatin damage in zebrafish and a rat model. The utility of the zebrafish model for discovering potentially otoprotective drugs is well documented. Owens et al. (2008), investigated a library of 10,960 compounds for otoprotective effects in zebrafish larvae and found that two benzothiophene carboximides, PROTO-1 and PROTO-2, acted as otoprotectants. A recent screen in the zebrafish lateral line of 10,000 small molecule inhibitors of cisplatin-mediated hair cell death showed that two compounds, cisplatin hair cell protectant 1 and 2 (CHCP1 and 2), may respectively reduce uptake of a cisplatin analog and affect an intracellular mechanism (Thomas et al., 2015b). Quinoline drugs protective against neomycin and gentamicin mediated damage have also been identified (Ou et al., 2012). Vlasits et al. (2012), discovered 10 compounds that prevent several aminoglycosides and cisplatin from damaging hair cells in the larval zebrafish lateral line. Evidently, fish models could provide a very productive framework in the future for screening drug molecules that could offer protection against ototoxic damage, but these drugs still need to be tested in mammalian models.

### Cell Cycle Mechanisms

Studies in zebrafish and mouse models demonstrate that they share key components of several hair cell proliferation pathways, e.g., Wnt, Notch and Rb (Hernández et al., 2007; Liu and Zuo, 2008; Ma et al., 2008; Moon et al., 2011; Head et al., 2013; Jacques et al., 2013; Lin et al., 2013; Mizutari et al., 2013; Wada et al., 2013). Despite this fact, hair cell proliferation does not normally occur in mammals after cells become post-mitotic (Burns and Corwin, 2013). One potential nexus for this divergent cellular behavior could involve regulation of the cell cycle (Liu and Zuo, 2008). Mammalian studies have identified a number of molecules that regulate hair cell entry into mitosis. One of these, the cell cycle inhibitor p27kip, can modulate mammalian hair cell regeneration (Chen and Segil, 1999; Löwenheim et al., 1999; Kanzaki et al., 2006; Ono et al., 2009; Oesterle et al., 2011; Liu et al., 2012; Walters et al., 2014). However, to this date, there is little information available on how cell cycle regulatory mechanisms function in fish hair cell regeneration or what environmental cues modulate them. Studies on this topic, and related signal transduction pathways, would be beneficial not only for our understanding of hair cell regenerative phenomena in fish, but could also have translational applications towards human health.

### Reinnervation

Reinnervation, the reestablishing of functional nervous system connections with target cells, is a necessary concomitant of hair cell regeneration. If neurons are unable to establish appropriate communication with new hair cells, then auditory and vestibular signaling to the brain will not occur. The neuromasts and associated neurons of the embryonic zebrafish lateral line can be stained facilitating the study of neural innervation of the neuromasts using mutant models (Raible and Kruse, 2000). Hair cell polarity in the zebrafish lateral line is important for establishing an appropriate sense of equilibrium (López-Schier et al., 2004). Mutant fish lacking a planar-polarity protein (Vangl2) have improperly oriented hair cells (López-Schier and Hudspeth, 2006). Nagiel et al. (2008), and Faucherre et al. (2009), have shown that PLL afferent neurons establish synaptic connections with regenerated hair cells with a particular orientation suggesting that they target a polarity guidance cue. Zebrafish unable to transduce mechanical stimuli have afferent neurons that exhibit increased arborization, less stability and more pathfinding errors (Faucherre et al., 2010). Intact lateral line hair cells are not required for neuronal regeneration, but in their absence deviation in pathfinding occurs (Villegas et al., 2012). Also, the synaptic ribbon protein, Ribeye, is required for synaptogenesis and afferent innervation in zebrafish (Sheets et al., 2011). Mo and Nicolson (2011), have investigated the role of N-ethylmaleimide-sensitive factor (Nsf) protein in hair cell synapse maintenance. Afferent neurons retract when Nsf decreases. Reduction of Nsf also caused brain-derived neurotrophic factor, BDNF, to accumulate suggesting that Nsf is required for BDNF release. Injection of BDNF only partially restores afferent synapses indicating that other signaling molecules may be involved in promoting hair cell synaptogenesis. Studies performed in mammalian models show that various stem cell populations can develop the features of spiral ganglion neurons and that their transplantation into the inner ear can restore some auditory function (Diensthuber et al., 2014; Géléoc and Holt, 2014). Another study transplanted spiral ganglion neurons into mice where these neurons had been destroyed and found that the replaced neurons reinnervated and formed new synaptic connections with hair cells (Martinez-Monedero et al., 2006). Additional studies in zebrafish will be needed to elucidate the mechanisms that modulate neuronal reinnervation with hair cells and their relatedness to mammalian organisms.

## Conclusion

Much has been learned in the last decade about hair cell death and regeneration in teleost fishes. Advances derived from studies in zebrafish and other fish species include understanding how ototoxins affect hair cells and discovering new otoprotectants that mitigate ototoxin damage, the role played by cellular proliferation and transdifferentiation during hair cell regeneration, and characterization of the cellular pathways involved in regeneration. Although the zebrafish model has the advantage of higher throughput and easier access to sensory hair cells than mammalian models, because of the different cellular and molecular characteristics of teleost and mammalian auditory cells, data obtained in zebrafish models may not apply to mammalian models. Thus, additional comparative studies involving zebrafish and mammal-based models will be needed to determine whether results obtained in teleosts can be translated into therapeutics to prevent or treat human hearing loss.

## Conflict of Interest Statement

The authors declare that the research was conducted in the absence of any commercial or financial relationships that could be construed as a potential conflict of interest.
